# Aerobic Physical Training Attenuates Oxidative Stress in the Spinal Cord of Adult Rats Induced by Binge-like Ethanol Intake

**DOI:** 10.3390/antiox12051051

**Published:** 2023-05-05

**Authors:** Amanda do Nascimento Rodrigues, Diane Cleydes Baia da Silva, Daiane Claydes Baia-da-Silva, Paulo Fernando Santos Mendes, Maria Karolina Martins Ferreira, Gabriel Sousa Rocha, Marco Aurelio M. Freire, Luanna Melo Pereira Fernandes, Cristiane do Socorro Ferraz Maia, Walace Gomes-Leal, Rafael Rodrigues Lima

**Affiliations:** 1 Laboratory of Functional and Structural Biology, Institute of Biological Sciences, Federal University of Pará, Belém 66075-110, Brazil; amanda.rodrigues@icb.ufpa.br (A.d.N.R.); diane.silva@ics.ufpa.br (D.C.B.d.S.); daiane.silva@ics.ufpa.br (D.C.B.-d.-S.); maria.ferreira@ics.ufpa.br (M.K.M.F.); 2 Graduate Program in Health and Society, Faculty of Health Sciences, State University of Rio Grande do Norte, Mossoró 59610-110, Brazil; gabrielrocha@alu.uern.br (G.S.R.); marcofreire@uern.br (M.A.M.F.); 3 Department of Morphology and Physiological Sciences, Center of Sciences Biological and Health, State University of Pará, Belém 66087-662, Brazil; 4 Laboratory of Pharmacology of Inflammation and Behavior, Health Sciences Institute, Pharmacy College, Federal University of Pará, Belém 66075-900, Brazil; crismaia@ufpa.br; 5 Laboratory of Experimental Neuroprotection and Neuroregeneration, Institute of Collective Health, Federal University of Western Pará, Santarém 68040-470, Brazil; wallace.leal@ufopa.edu.br

**Keywords:** ethanol, binge drinking, moderate physical activity, redox system, spinal cord

## Abstract

Binge drinking is the most frequent consumption pattern among young adults and remarkably changes the central nervous system; thus, research on strategies to protect it is relevant. This study aimed to investigate the detrimental effects of binge-like EtOH intake on the spinal cord of male rats and the potential neuroprotective effects provided by moderate-intensity aerobic physical training. Male Wistar rats were distributed into the ‘control group’, ‘training group’, ‘EtOH group’, and ‘training + EtOH’. The physical training protocol consisted of daily 30-min exercise on a treadmill for 5 consecutive days followed by 2 days off during 4 weeks. After the fifth day of each week, distilled water (‘control group’ and ‘training group’) or 3 g/kg of EtOH diluted at 20% w/v (‘EtOH group’ and ‘training + EtOH group’) was administered for 3 consecutive days through intragastric gavage to simulate compulsive consumption. Spinal cord samples were collected for oxidative biochemistry and morphometric analyses. The binge-like EtOH intake induced oxidative and tissue damage by decreasing reduced glutathione (GSH) levels, increasing lipid peroxidation (LPO), and reducing motor neurons (MN) density in the cervical segment. Even under EtOH exposure, physical training maintained GSH levels, reduced LPO, and prevented MN reduction at the cervical segment. Physical training is a non-pharmacological strategy to neuroprotect the spinal cord against oxidative damage induced by binge-like EtOH intake.

## 1. Introduction

Easy access to and abuse of ethanol (EtOH) have been associated with health and social harm in several countries; thus, this psychotropic drug figures as a global public health concern [1,2,3]. The excessive consumption of alcoholic beverages in 2010 cost the United States approximately $249 billion, of which 77% was related to binge drinking [4]. The EtOH abuse can result in cognitive disturbances, depressive episodes, severe anxiety, insomnia, liver and kidney disorders [5,6,7]. Interestingly, these detrimental effects are directly proportional to the type and duration of EtOH consumption [8,9,10]. In addition, excessive EtOH consumption has been related to dopamine neurotoxic effects through the increase of α-synuclein in rats and accumulation of Aβ and Tau phosphorylation in humans [11].

Heavy episodic drinking (binge), in which the EtOH concentration reaches at least 0.08 g per deciliter of blood, has grown significantly among adolescents and young adults [12,13,14,15,16,17,18,19,20]. In animal studies, binge-like EtOH consumption has been associated with tissue changes and oxidative stress in salivary glands [21,22], hippocampus, and prefrontal cortex, damage of motor and cognitive functions [23,24,25], and decrease of alveolar bone quality [26,27]. One of the main harmful effects of EtOH is the increase of reactive oxygen species (ROS) and decrease of antioxidants such as the glutathione peroxidase (GPx) enzyme [28,29].

Among the strategies to reduce these detrimental effects, physical training has been associated with neuroinflammation reduction, improvement of cognitive functions, an increase of brain-derived neurotrophic factor (BDNF) levels, modulation of neurogenesis, and cerebral oxidative stress [30,31,32,33,34,35].

Muscle contraction releases myokines in the bloodstream such as peroxisome proliferator-activated receptor coactivator 1-alpha (PGC-1α) and the nuclear factor erythroid 2-related factor 2 (Nrf2) that acts as a transcription factor for antioxidant enzymes in different tissues [36]. Chronic physical training seems to increase the expression of the important antioxidant markers superoxide dismutase 1 (SOD1), reduced glutathione (GSH), and GPx [37].

Our research group has shown that training on a treadmill attenuated the EtOH-induced detrimental effects in terms of tissue and functional changes in the cerebellum as well as hippocampal functional changes [19,38]. In comparison to anaerobic training, aerobic training substantially improves the performance of executive control processes [39].

The relation between the neuroplasticity benefits of physical training and the detrimental effects of EtOH consumption is not completely elucidated. The mechanisms by which physical training can improve the reflexes associated with the spinal cord of alcoholics are not established. This study aimed to investigate the detrimental effects of binge-like EtOH intake on the spinal cord of male rats and the potential neuroprotective effects provided by moderate-intensity aerobic physical training. 

## 2. Materials and Methods

### 2.1. Ethical Aspects and Experimental Animals

This study was approved by the Ethics Committee on Animal Experimentation of the UFPA (license number 1423181219) and followed both ARRIVE 2.0 guideline and NIH Guide for the Care and Use of Laboratory Animals [40]. Sixty male Wistar rats (90 days old; weighing between 172 to 199 g) were maintained in collective cages (4 animals each) with water and food ad libitum. The cages were housed in a climate-controlled room (~25 °C) with a 12 h light/dark cycle (lights on 7 a.m.).

### 2.2. Exposure Protocol and Experimental Groups

Only male rats were selected to allow direct comparisons with our previous findings [19,38] and to avoid bias related to gender variability in physical training performance and alcohol metabolism [41].

**Figure 1:**
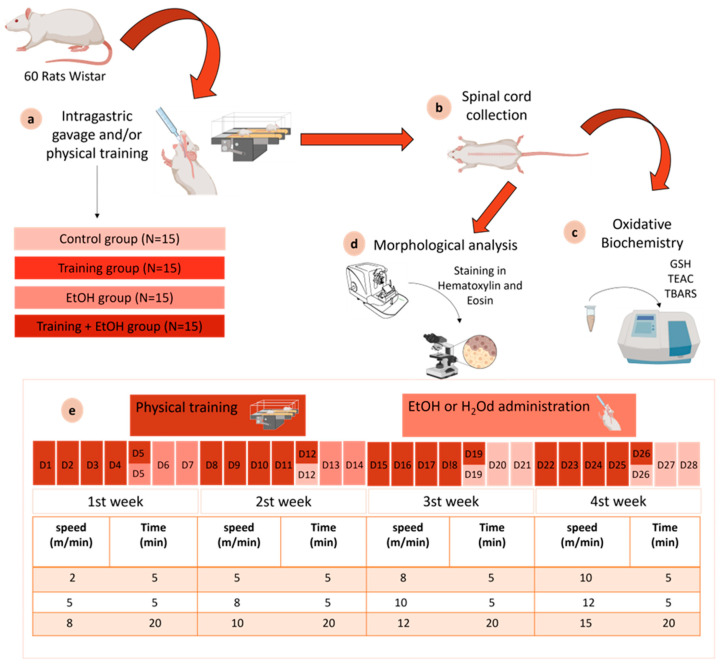
Study design. (**a**) experimental groups; (**b**) collection of biological material (spinal cord sectioned into regions: cervical, thoracic, and lumbar); (**c**) Oxidative biochemistry analysis (GSH, TEAC, and TBARS); (**d**) Morphological analysis by counting motoneurons in Hematoxylin and Eosin (HE); (**e**) Details of exposure to EtOH or physical training during the experimental period.

The animals were randomly distributed into 4 groups (*n* = 15): ‘control group’ (sedentary animals treated with distilled water), ‘training group’ (animals submitted to physical training and treated with distilled water), ‘EtOH group’ (sedentary animals treated with EtOH), and ‘training + EtOH group’ (animals submitted to physical training and treated with EtOH) (Figure 1).

#### 2.2.1. Physical Training Protocol

The group allocation was adapted from Arida et al. 2007 [42], in which the animals were subjected to training on a treadmill for 3 days (10 min/day at a speed of 8 m/min and 0° of inclination) and the performance of each animal was classified as 1 = refused to run; 2 = below average runner (stops and runs in the wrong direction); 3 = average runner; 4 = above average runner; 5 = good runner (consistently stayed at the front of the treadmill). The animals classified as good runners were selected for the ‘training group’ and ‘training + EtOH group’.

The physical training protocol was adapted by Lamarão et al. 2019 [19] and Pamplona et al. 2019 [38], in which the animals were daily subjected to 30-min training on the treadmill with progressive speed increase for 5 consecutive days followed by 2 days off during 4 weeks [19,38] (Figure 1). All animals successfully performed the training protocol without accidental injuries, infarction, fatigue, and lack of willingness to exercise [42].

#### 2.2.2. Drinking Protocol

After the fifth day of each week, distilled water (‘control group’ and ‘training group’) or 3 g/kg of EtOH diluted at 20% *w*/*v* (‘EtOH group’ and ‘training + EtOH group’) was administered for 3 consecutive days through intragastric gavage. The animals were weekly weighted to adjust the dose. The binge-like EtOH administrations aimed to simulate a pattern of compulsive consumption for 4 weeks [22,24,43].

### 2.3. Euthanasia and Spinal Cord Collection

The animals were anesthetized through the injection of ketamine hydrochloride (90 mg/kg) and xylazine hydrochloride (9 mg/kg). After the absence of corneal reflex, the spinal cord samples of 6 animals per group were collected, cleaned, and stored at −80 °C for oxidative biochemistry analyses. 

For the morphological analysis, the spinal cord was removed after transcardial perfusion with 0.9% heparinized saline solution followed by 4% paraformaldehyde [44] and then divided into cervical, thoracic, and lumbar segments.

### 2.4. Oxidative Biochemistry Analyses

Tissues were thawed and ultrasonically homogenized in Tris-HCl buffer (20 mM, pH 7.4) at 4 °C and 1:10 ratio. The tissue preparation was detailed in our previous study [45].

#### 2.4.1. Protein Concentration Assay

The determination of total protein levels followed the method proposed by Bradford (1976) [46], in which the proteins bind to the Coomassie brilliant blue dye and form a blue compound with maximum absorbance at 595 nm.

#### 2.4.2. Measurement of Trolox Equivalent Antioxidant Capacity (TEAC)

TEAC levels were determined through the colorimetric method described by Miller et al. (1993) [47] e modified by Re et al. (1999) [48]. Briefly, the reaction between 2,2-azinobis [3-ethylbenzothiazoline-6-sulfonic acid] diammonium salt (ABTS) and potassium persulfate (K_2_S_2_O_8_) produces the blue/green ABTS•+ chromophore. The antioxidants present in the sample to this preformed radical cation reduce it to ABTS on a time scale depending on the antioxidant capacity, concentration of antioxidants, and duration of the reaction. The reaction was spectrophotometrically measured throughout 5 min by observing the absorbance change at 734 nm. Total antioxidant capacity was expressed in μmol/g of protein.

#### 2.4.3. Measurement of Reduced Glutathione (GSH)

The ability of GSH to reduce 5,5-dithiobis-2-nitrobenzoic acid (DTNB) to nitrobenzoic acid (TNB) was quantified by spectrophotometry at 412 nm. This method was adapted from Ellman (1959) [49] and the GSH concentration was expressed as μg/g of protein.

#### 2.4.4. Determination of Thiobarbituric Acid Reactive Substances (TBARS)

Lipid peroxidation (LPO) was estimated through the formation of the malondialdehyde with thiobarbituric acid (MDA-TBA) complex with pH 2.5 at 94 °C [50]. The samples were read at 535 nm and the results were expressed in nM/g of protein.

### 2.5. Morphometric Analysis

The spinal cord samples were post-fixed in Bouin’s solution for 12 h, dehydrated in increasing alcohol solution, clarified in xylene, and embedded in paraplast (McCormick Scientific; Saint Louis, MO, USA). Subsequently, 7-μm-thick cross-sections of the cervical, thoracic, and lumbar segments were obtained, mounted on microscopy slides, stained with hematoxylin and eosin (HE), and coverslipped with mounting medium (Entellan; Merck, Darmstadt, Germany). The samples were observed under a brightfield optical microscope (Nikon Eclipse Ci H550; Nikon, Tokyo, Japan) equipped with a digital camera (DS-Fi3; Nikon, Tokyo, Japan) to determine the motor neurons (MN) density.

MN quantification followed the protocol proposed by Ferucci et al. (2018) [51], who described these cells located in the ventral horn of the spinal cord with basophilic characteristics and with poorly condensed chromatin nuclei. MN counting was performed at different fields of the ventral horns of the cervical, thoracic, and lumbar segments with the aid of the NIH ImageJ software version 1.52 (http://rsb.info.nih.gov/ij/ (accessed on 15 February 2023)).

### 2.6. Statistical Analyses

The normal distribution of the data was verified by the Shapiro-Wilk test (GraphPad Prism 8.0.2; GraphPad Software Inc., San Diego, CA, USA). The body weight curve was evaluated by using two-way ANOVA followed by the Tukey post hoc test. Oxidative biochemistry and MN density were analyzed by using one-way ANOVA, partial eta-squared (η^2^) analysis, and the Tukey post hoc test. The results were expressed in mean ± standard error of the mean (SEM), values of *p* ≤ 0 05 were considered significant, and the partial η^2^ analysis was considered: minimal to no effects (η^2^ between 0.00 and 0.29), small effects (η^2^ between 0.30 and 0.50), moderate effects (η^2^ between 0.50 and 0.70), and large (η^2^ between 0.71 and 1.00) [52].

## 3. Results

### 3.1. Body Weight Gain Was Not Influenced by Binge-like EtOH Intake and Physical Training 

In all groups, a significant body weight gain was observed from baseline up to 4 weeks (Control group: 177.65 ± 0.99 vs. 192.97 ± 1.46, *p* < 0.0001; training group: 178.85 ± 1.89 vs. 194.22 ± 1.56, *p* < 0.0001; EtOH group: 179.50 ± 1.33 vs. 191.83 ± 2.09; *p* = 0.0003; training + EtOH group: 178.67 ± 2.15 vs. 193.18 ± 2.21, *p* = 0.0007). 

**Figure 2:**
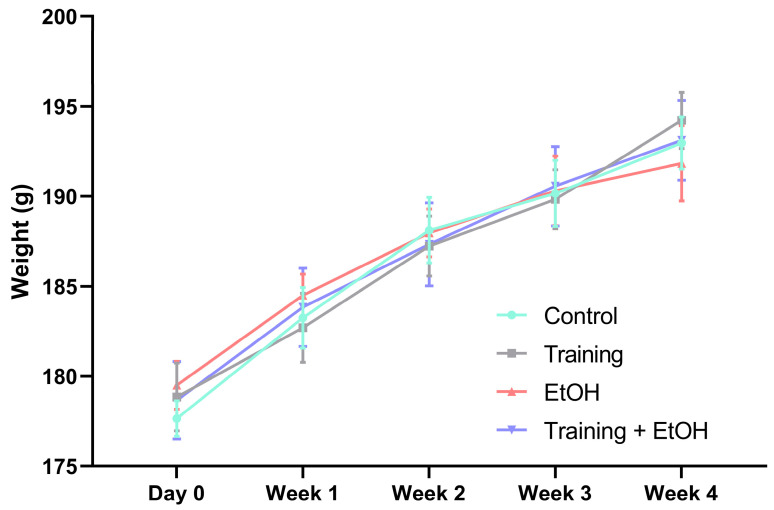
Effect of binge-like EtOH intake and/or training on body weight. There was no significant difference among groups (*n*= 15). Results are expressed as mean ± standard error of the mean. Two-way ANOVA and Tukey post hoc test (*p* < 0.05).

There was no significant difference in body weight mean among groups after 4 weeks (Control: 192.97 ± 1.46; training: 194.22 ± 1.56; EtOH: 191.83 ± 2.08; training + EtOH: 193.18 ± 2.21) (Figure 2). Regular physical training and/or binge-like EtOH intakes did not significantly alter the body weight gain of the animals (Figure 2).

### 3.2. Regular Physical Training Attenuated EtOH-Induced Oxidative Stress in the Spinal Cord of Rats

**Figure 3:**
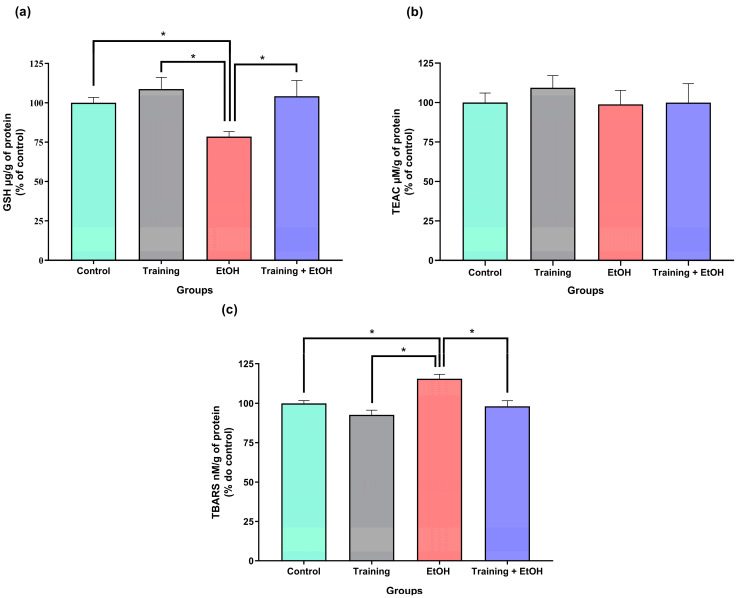
Oxidative biochemistry analyses (*n* = 6). (**a**) GSH; (**b**) TEAC; (**c**) TBARS. Results are expressed as a percentage (%) of control (mean ± standard error of the mean). Asterisks (*) indicate significant differences (*p* < 0.05). One-way ANOVA test followed by Tukey post hoc test.

Binge-like EtOH intake caused a significant decrease in GSH levels (EtOH group: 78.51 ± 3.18%) when compared to the ‘control group’ (100.00 ± 3.38%; *p* = 0.0243; η^2^ = 0.964), ‘training group’ (108.70 ± 7.44%; *p* = 0.0014; η^2^ = 1.368), ‘training + EtOH group (104.20 ± 4.07%; *p* = 0.0063; η^2^ = 0.761). These data indicate that physical training minimized the changes induced by EtOH (Figure 3a).

Regardless of physical training and/or binge-like EtOH intake, TEAC levels were not significantly different among groups (‘control group’: 100.00 ± 6.03%; ‘training group’: 109.40 ± 3.15%; ‘EtOH group’: 98.83 ± 3.60%; ‘training + EtOH group’: 99.95 ± 5.38%; *p* > 0.05; Figure 3b).

Binge-like EtOH intake significantly increased TBARS levels (114.9 ± 2.7%) when compared to the other groups (‘control group’: 99.94 ± 1.77%; *p* = 0.0039; η^2^ = 1.580; ‘training group’: 92.67 ± 2.96; *p* = 0.0001; η^2^ = 1.227; ‘training + EtOH group’: 98.07 ± 3.58%; *p* = 0.0013; η^2^ = 1.113; Figure 3c).

### 3.3. Regular Physical Training Did Not Prevent MN Density Reduction in the Cervical Segment Induced by Repeated Binge-like EtOH Intake

**Figure 4:**
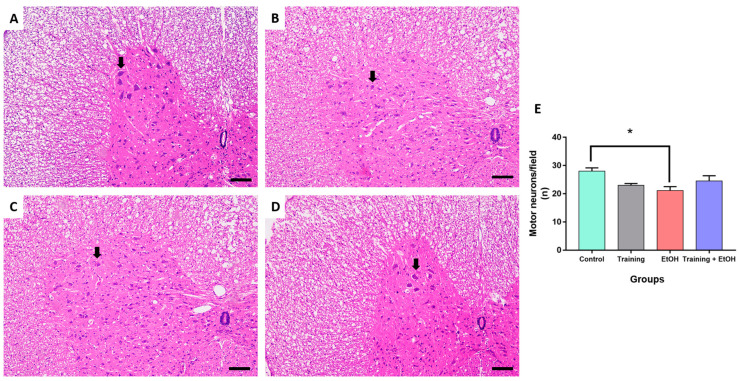
Representative HE-stained photomicrographs of the cervical segment of the spinal cord of rats (100μm scale bar). (**A**) control group; (**B**) training group; (**C**) EtOH group, and (**D**) training + EtOH group. (**E**) Graph of MN density in each group (*n* = 9) expressed as mean ± standard error of the mean of the number of cells counted per field. Black arrows indicate motor neurons. Asterisk (*) indicates a significant difference (One-way ANOVA and Tukey post hoc test, *p* < 0.05).

**Figure 5:**
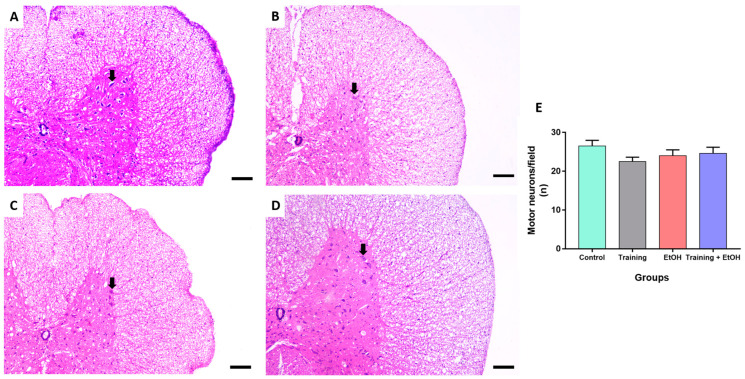
Representative HE-stained photomicrographs of the thoracic segment of the spinal cord of rats (100μm scale bar). (**A**) control group; (**B**) training group; (**C**) EtOH group, and (**D**) training + EtOH group. (**E**) Graph of MN density in each group (*n* = 9) expressed as mean ± standard error of the mean of the number of cells counted per field. Black arrows indicate motor neurons.

**Figure 6:**
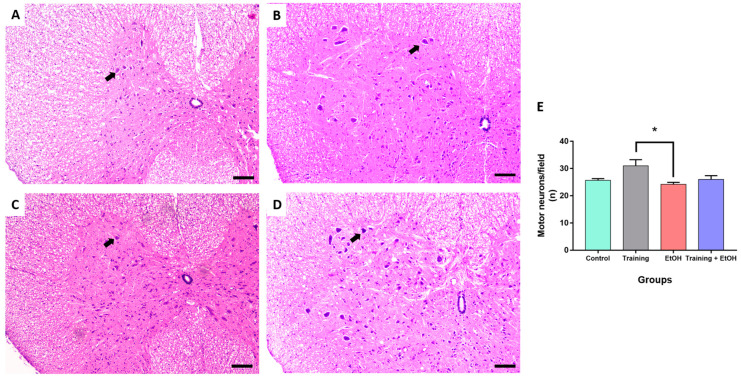
Representative HE-stained photomicrographs of the lumbar segment of the spinal cord of rats (100μm scale bar). (**A**) control group; (**B**) training group; (**C**) EtOH group, and (**D**) training + EtOH group. (**E**) Graph of MN density in each group (*n* = 9) expressed as mean ± standard error of the mean of the number of cells counted per field. Black arrows indicate motor neurons. Asterisk (*) indicates a significant difference (One-way ANOVA and Tukey post hoc test, *p* < 0.05).

Binge-like EtOH intake significantly reduced the MN density in the cervical segment only when compared to the control group (‘control group’: 28 ± 1.134; ‘EtOH group’: 21.11 ± 1.419; *p* = 0.011; η^2^ = 0.188) (Figure 4). The MN density in the thoracic segment was not significantly different among groups (*p* > 0.05) (Figure 5). In the lumbar segment, the MN density of the ‘training group’ was significantly different in comparison to ‘EtOH group’ (‘training group’ 31 ± 2.236; ‘EtOH group’ 24.22 ± 0.702; *p* = 0.061; η^2^= 0.576); however, this difference was not significant when training was associated with binge-like EtOH intake (‘training + EtOH group’) (Figure 6).

## 4. Discussion

This study evaluated the biochemical and morphological effects in the spinal cord of rats subjected to physical training and/or binge-like EtOH intake for 4 weeks as well as potential neuroprotective effects induced by physical training. The results showed that binge-like EtOH intake decreased GSH levels and increased LPO (estimated through TBARS evaluation); in addition, physical training was able to protect the spinal cord against EtOH-induced oxidative damage. The EtOH-induced reduction of MN density in the cervical segment may be explained by their damage susceptibility to ROS, as observed in lateral amyotrophic sclerosis [53]; in addition, physical training was not able to avoid these morphological changes.

The spinal cord is adjacent to the cerebellum and extends from the medulla oblongata to the lower edge of the first lumbar vertebra [54]. This organ contains several cell types (astrocytes, oligodendrocytes, microglia, and MN) that receive motor information from the brain and sensory information from the body [55]. The spinal cord may be sensitive to neuronal damage induced by EtOH binge drinking. Therefore, this original study evaluated the effects of repeated binge-like EtOH intake in the spinal cord and the potential protective effect provided by physical training against oxidative and morphological damage.

Binge drinking is the excessive consumption of alcohol in a short period of time that leads blood alcohol concentration of 0.8 g/L or above [56]. EtOH is metabolized through alcohol dehydrogenase (ADH), catalase, and microsomal EtOH-oxidizing system (MEOS; CYP2E1) [57]. The high EtOH intake increases the expression and activity of MEOS, which in turn generates the production of acetaldehyde through the formation of ROS, such as hydrogen peroxide (H_2_O_2_) [58]. Acetaldehyde is the most toxic metabolite resulting from alcohol metabolism since it causes DNA mutations and chromosomal damage, upregulates CYP2E1 expression, and increases oxidative stress [58,59]. Subsequently, this system produces additional ROS that may damage mitochondria, and generate cytotoxicity, inflammation, and cell death [57,60,61]. The oxidative imbalance and tissue damage caused by EtOH have been evidenced in several regions of the central nervous system, such as the hippocampus [24,38,62] and cerebellum [19].

EtOH induces detrimental effects through different mechanisms, such as excitotoxicity, neuroinflammation, and oxidative stress [21]. A study on prenatal alcohol intake has shown that the number and the morphology of MN were significantly reduced. The authors highlighted the detrimental effects of EtOH (neurotoxicity and oxidative stress) in this critical period of the development of the nervous system [63].

Alcohol intake induces oxidative stress through the imbalance between the antioxidant system and ROS production and ultimately leads to cell dysfunction [64]. This study evaluated the oxidative stress pathway since the EtOH metabolism overproduces ROS [65,66]. Therefore, this study evaluated the imbalance in the redox system through the quantification of GSH and total antioxidant capacity; in addition, MDA was quantified to estimate the LPO induced by the ROS increase. The results of this study demonstrated that EtOH binge drinking induces oxidative damage, decreases GSH levels, and increases LPO; in addition, physical training prevents the reduction of this antioxidant and reduces LPO.

Our research group has previously evaluated the effect of EtOH on the central nervous system by using the same experimental model. Pamplona et al. (2019) [38] observed that EtOH binge drinking decreased GSH levels by 26.42% and increased TBARS levels by 50.11% in the hippocampus of rats; in addition, moderate-intensity physical training maintained GSH levels and increased LPO. Lamarão-Vieira et al. (2019) [19] evaluated the effects of EtOH in the cerebellum and reported an increase of 748.30% in TBARS levels without a reduction in GSH levels. Therefore, this study findings regarding the oxidative effects of EtOH on the central nervous system corroborate with Pamplona et al. (2019) [38] and Lamarão-Vieira et al. (2019) [19], albeit these studies demonstrated different susceptibilities among regions.

Although a significant decrease in GHS levels was induced by binge-like EtOH intake, physical training did not lead to significant changes in the antioxidant capacity.

Overall, regular moderate physical training definitely improves muscle tone, cardiovascular function, brain processes such as cognition and memory, and quality of life. Moreover, physical training significantly controls ROS-induced oxidative by up-regulating endogenous antioxidant defenses [67] and inducing neuroplasticity [68]. Among the neuroprotective mechanisms induced by physical training, the Nrf2 signaling pathway is very efficient in attenuating cellular damage caused by neurotoxic substances. Tsou et al. (2015) demonstrated the neuroprotective effect of physical training in rats exposed to 1-methyl-4-phenylpyridine (MPP^+^), which is the major bioactive and toxic metabolite of MPTP. The animals submitted to physical training had a lower loss of nigral dopaminergic neurons than sedentary animals; however, Nrf2 knockout animals had similar responses to MPP^+^ irrespective of physical training [69]. More specifically, one study evaluated the effect of injecting cholera toxin-conjugated saporin to selectively kill the MN of the vastus medialis muscles of rats. The authors observed that the dendritic loss of animals subjected to physical training was significantly lower than that of sedentary animals, which is fundamental for the communication and survival of MN [70].

Physical training can be individualized by specific frequency, type, and intensity [71]. This study followed the method proposed by Arida et al. (2007) [42] and adapted by Lamarão-Vieira et al. (2019) [19], in which the animals were subjected to physical training with progressive intensity during 4 weeks and 4 cycles of binge-like EtOH intake (3 g/kg/day, 20% *w*/*v*). The results demonstrated that physical training and/or binge-like EtOH intake did not significantly change the weight of the animals since the weight growth curves were similar among groups.

The exposure of the rats to a regular and progressive physical training protocol aimed to induce a compensatory response (also known as hormesis) to exercise-induced oxidative stress and/or another condition such as EtOH exposure [71,72]. Our research group recently demonstrated that binge-like EtOH intake promoted oxidative and functional changes in the cerebellum [19] and hippocampus [24,38,62] of rats as well as the protective effect provided by physical training.

Therefore, regular physical training induced an adaptive response by increasing GSH levels, improving the antioxidant defense against the radicals produced during training, and protecting the spinal cord against the free radicals produced by EtOH metabolism. In this study, the positive modulation of the redox system induced by physical training was observed by the increase in GSH levels, which is the main antioxidant defense mechanism against EtOH [73].

The investigation of other markers of systemic oxidative stress such as 8-iso-prostaglandin F2α (8-iso-PGF2α), sp-NOX2, proteomics, and 8-hydroxyguanosine may reveal underlying mechanisms not elucidated in this study. The 8-iso-PGF2α is formed by the peroxidation of arachidonic acid in lipids and has been shown as a good marker of oxidative damage [74]. Nicotinamide adenine dinucleotide phosphate oxidase2 (NOX2) increases ROS formation and releases anti-inflammatory molecules [75,76].

This study determined the MN density in the ventral horn of the spinal cord, which is directly related to motor activity [54]. The binge-like EtOH intake protocol reduced the MN density in the cervical segment when compared to the control group, albeit no remarkable differences were observed in the thoracic and lumbar segments. Nevertheless, physical training did not provide significant protection to spinal cord cells against EtOH exposure. It may be explained by the need for longer survival times to provide neuroprotection for the MN after oxidative stress reduction, which is an acute pathological event observed in several acute and chronic neural disorders. Moreover, several populations of short- and long-range projection interneurons are also found in the spinal cord and are likely to differently respond to EtOH intoxication. Physical training may also provide neuroprotection to other cell populations rather than MN. For instance, propriospinal interneurons play a key role to synchronize motor activity and ambulation of the spinal cord [77,78] and may distinctly respond to both EtOH-induced detrimental effects and physical training-induced neuroprotective effects investigated in this study. Therefore, these hypotheses are encouraged to be addressed in further studies.

Although physical training times were standardized for all animals, it must be emphasized that the intensity can be modulated by the maximum volume of oxygen; therefore, further studies that take into account the individual physical training capacity may more accurately determine the modulating effects to attenuate EtOH-induced damage. Furthermore, novel studies should investigate the response of different cell populations of the spinal cord such as propriospinal interneurons to EtOH intoxication as well as the potential neuroprotective effects of physical training.

## 5. Conclusions

Repeated cycles of binge-like EtOH intake caused an oxidative imbalance in the spinal cord of rats by decreasing GSH levels, increasing LPO, and reducing MN density at the cervical segment. Physical training figures as a valuable tool to restore oxidative balance since it maintained GSH levels and reduced LPO levels even under EtOH exposure. Moderate aerobic physical training is a non-pharmacological strategy to neuroprotect the spinal cord against oxidative damage induced by binge-like EtOH intake.

## Data Availability

All data are available within the article.
